# Expanding Pharmaceutical Access Via Over the Counter Drugs

**DOI:** 10.1007/s43441-024-00709-3

**Published:** 2024-10-20

**Authors:** Terra Marie M. Jouaneh, Vrushab Gowda, Brian J. Miller

**Affiliations:** 1 Department of Medicine, Johns Hopkins University School of Medicine, Baltimore, MD 21287 USA; 2 Massachusetts General Hospital, Boston, MA 02114 USA; 3 Harvard Medical School, Boston, MA 02115 USA; 4 American Enterprise Institute, Washington, DC 20036 USA

## Abstract

This commentary addresses the recent U.S. Food & Drug Administration (FDA) proposed rule to expand access to nonprescription drugs through additional conditions of nonprescription use (ACNU). It surveys the various pathways to market for pharmaceutical products, noting the distinct requirements for over-the-counter (OTC) products differentiating them from prescription products. It subsequently reviews the ACNU proposed rule, weighing its potential benefits against possible limitations. With a view towards the future, the ACNU proposed rule is acknowledged as part of a longstanding tradition to expand drug channels in a risk-stratified fashion with increasing clinical oversight to address in tandem increasing consumer risks. Finally, the proposed rule also serves as a potential prelude for a future behind the counter drug pathway.

It is time to think differently about treating chronic disease. According to the Centers for Disease Control and Prevention, over 120 million Americans suffer from inadequately treated hypertension or diabetes. The latter alone contributes to over $327 billion in direct medical costs and lost productivity [1], a figure compounded by an insufficient pool of physicians and advanced practitioners to guide treatment.

Building upon existing longstanding efforts to support access to nonprescription drugs, separately both direct-to-consumer sales and pharmacist-mediated medication use offer unique levers to increase the number of drug channels thus expanding access to pharmaceuticals. This viewpoint reviews the current paths to market for pharmaceutical products, evaluates the U.S. Food and Drug Administration’s (FDA) recent proposed rule expanding nonprescription over-the-counter (OTC) drugs through Additional Condition for Nonprescription Use (ACNU) [2], and explores the potential for a future behind-the-counter (BTC) pathway to expand basic medication access for a variety of societal needs such as the treatment of chronic diseases.

## Existing Paths to Market for Pharmaceutical Products

Prior to receiving marketing authorization from the FDA, all developers of pharmaceutical products must provide substantial evidence of their safety and efficacy [3]. Depending on their marketing pathway, the FDA may impose additional requirements. Prescription drugs must be preceded by a clinician’s order as their use often demands a clinician’s direct management, monitoring, instruction, or oversight. In contrast, nonprescription products are assessed with additional label comprehension, self-selection, and other consumer studies. With the appropriate Drug Facts Label (DFL), these products are approved for consumer self-administration without the need for a prescriber [4].

While the Federal Food, Drug, and Cosmetic Act defines two formal drug classifications – OTC and prescription – some drugs sit at the nexus of the two, effectively placing the pharmacist as the key intermediary between patients and medications [5]. Drugs containing codeine and nonprescription insulin are among several BTC drugs requiring some element of pharmacist consultation prior to dispensing. This contrasts with other jurisdictions, Australia among them, which have a formal class of “pharmacist-only medications.” These BTC drugs encompass a range of medications including albuterol inhalers for asthma treatment and moderate or high-potency topical steroids like triamcinolone, currently available only via prescription in the US.

The public relies upon a wide range of nonprescription medications for self-management of minor ailments, pain relief, fever reduction, allergy control, and cough suppression, among others, offering convenience and immediate relief. The DFL on each medication’s packaging details its active ingredients, uses, warnings, directions, and other pertinent information on the outside container to ensure safe and effective use. However, space on the DFL is limited, potentially impeding adequate communication of all product information necessary for informed consumer self-selection [6].

## Introducing ACNU: a Revolutionary Middle Ground

In 2022, the FDA proposed an expansion of the OTC marketplace through the use of ACNU. This proposed rule, if finalized, would establish additional requirements for a new pathway for certain drugs to be marketed as nonprescription products. It was drafted intentionally broadly to promote creative submissions, which can include additional labeling requirements or technology-enabled, consumer-facing tools. The proposed rule would require sponsors to submit a statement justifying the ACNU’s necessity and details of its operationalization, both backed by adequate studies. The FDA has provided several examples, such as an ACNU requiring a consumer to complete a self-selection questionnaire via a mobile application or pharmacy kiosk in order to purchase a nonprescription product. One can imagine a patient with hyperlipidemia self-selecting and obtaining a statin by inputting recent lipid lab results into an application, thereby promoting autonomy and broadening access to chronic disease management. This is already a reality that manufacturers are working to execute, such as through the Technology-Assisted Cholesterol Trial in Consumers (NCT04964544) [7].

The proposed rule would apply to both new drug applications and abbreviated new drug applications for branded and generic nonprescription drugs. Notably, nonprescription drugs currently marketed under an approved application do not require an ACNU as the FDA has previously determined that labeling alone is sufficient for safe and effective use. In the proposed rule, the FDA posited that a product approved as both a prescription drug and a nonprescription drug with an ACNU could be simultaneously marketed because the ACNU provides a meaningful difference between the two products. Simultaneous marketing gives patients greater flexibility in obtaining drugs by either utilizing their health care provider for a prescription or fulfilling the requirements of the ACNU, obtaining the drug without a prescription.

Simultaneous marketing represents a change from historical practice and prior FDA policy. In some cases, OTC and prescription products have remained distinct by marketing different molecular moieties within a pharmacologic class (e.g., pantoprazole is only available as a prescription while omeprazole is available OTC). In other cases, simultaneous marketing is avoided via differing formulations or dosages with one product available OTC and another available as a prescription (e.g. hydrocortisone cream 1% versus 2.5% or omeprazole 20 mg versus 40 mg).

Manufacturers have expressed concerns with simultaneous marketing [8], specifically consumer confusion – a concern yet to be fully documented in research trials – and attribution errors in adverse event reporting. Regardless of which view of simultaneous marketing prevails, policy change will only be actualized once FDA approves an industry-submitted application.

OTC products with an ACNU may present some limitations regarding patient access to technology such as the prevalence of broadband internet at home or in-pharmacy, smartphone ownership and usage, or the availability of pharmacies with appropriate kiosks to serve as alternatives to consumer smartphone use. Recent evidence is supportive of a narrowing technological divide by age and socioeconomic status, with a recent survey demonstrating that 86% of Medicaid beneficiaries own smartphones [9] while 26.3% of Medicare beneficiaries lacked access to either a smartphone with a wireless data plan or a home computer with high-speed internet [10]. In the remaining gap, independent and chain store pharmacies will play an important role in promoting access to OTC ACNU products.

Despite ACNU, some medications may still require more stringent monitoring by a healthcare provider and thus remain as prescription-only products. OTC ACNU may result in fewer nonprescription-only drugs if FDA increasingly requires ACNU as a regulatory component for OTC drugs, a slippery slope in risk mitigation. Finally, in light of the landmark 2024 *Loper Bright Enterprises v. Raimondo* Supreme Court decision repealing Chevron deference, if finalized an OTC ACNU rule could be challenged and subject to judicial review.

## A Sliding Scale to Promote Safety

The ACNU proposed rule is a long-delayed step in implementing former FDA commissioner Scott Gottlieb’s commitment to empower consumer choice and further access to nonprescription drugs [11], particularly for those who face geographic or socioeconomic limitations to accessing primary or specialty care. Economic benefits include both potential decreased medical service costs and insurance administrative waste. Moreover, by recognizing the benefits of more drug channels coupled with increasing clinician involvement, ACNU creates more opportunities for access to basic medications for those who might otherwise lack access.

The creation of new drug channels may establish an early policy precedent for studying the potential of a later BTC drug class that leverages the role of pharmacists as a key learned intermediary [12]. Further research is needed in order to expand upon prior limited research efforts [13] and to better explore the risks and benefits of prescription to BTC switches in other markets, analogous to prior work examining prescription to OTC switches [14].

While the framework suggested in this proposal does not statutorily establish a third, formal channel for access to pharmaceutical products, it shifts the policy discussion towards thinking about cultivating a range of drug channels in tandem with increasing clinical oversight: OTC, OTC with ACNU, BTC, and prescription products. The ACNU proposal can be utilized as a lever to both (1) advance pharmacy practice beyond their current policing action for the handful of existing BTC drugs, as well as (2) expand patient access to low-cost, consumer-facing products.

Broader regulatory risks remain. For example, FDA risk tolerance and review practices could shift with the creation of more channels, functionally shunting new OTC products into the OTC ACNU class or even a potential future BTC class. Changes in policy could occur through explicit guidance or rulemaking, or alternatively through unwritten, more difficult to characterize practices such as increased reviewer-level product denials of OTC new drug applications. Finally, consumer and clinician barriers remain, with OTC ACNU potentially placing technological barriers on consumers, while a separate potential expanded BTC drug channel would place burdens on community pharmacists.

## Conclusion

Addressing widespread and persistent shortcomings in chronic disease management necessitates innovative policy approaches to expand basic drug access. The FDA’s proposal for an OTC class expansion through the ACNU proposed rule despite its imperfections offers a pragmatic policy solutions to expand basic medication access. OTC ACNU and other potential policy solutions expanding drug channels such as a behind-the-counter (BTC) pathway offer the opportunity to un-gate the pharmaceutical industry to innovate and expand access. These efforts, worthy of further exploration and study by policymakers and the FDA alike, reflect a shift towards more flexible delivery models aimed at improving outcomes through expanded access to basic medications.

## Data Availability

No datasets were generated or analysed during the current study.
